# Increased lysophosphatidylcholine acyltransferase 1 expression is unrelated to prognosis of esophageal cancer patients

**DOI:** 10.1007/s00432-021-03686-4

**Published:** 2021-06-19

**Authors:** Eugen Bellon, Katharina Grupp, Tarik Ghadban, Michael Tachezy, Kai Bachmann, Jakob Robert Izbicki, Ronald Simon, Guido Sauter, Claudia Hube-Magg, Nathaniel Melling

**Affiliations:** 1 General, Visceral and Thoracic Surgery Department and Clinic, University Medical Center Hamburg-Eppendorf, Hamburg, Germany; 2 Institute of Pathology, University Medical Center Hamburg-Eppendorf, Hamburg, Germany

**Keywords:** LPCAT1, Tissue microarray, Esophageal cancer

## Abstract

**Introduction:**

Lysophosphatidylcholine acyltransferase 1 (LPCAT1) has repeatedly been suggested to be associated with tumorigenesis. To evaluate the role of LPCAT1 in esophageal cancer, LPCAT1 immunostaining was analyzed on a tissue microarray containing samples from esophageal cancer patients.

**Results:**

In benign esophageal tissue, LPCAT1 staining was detectable in low intensities. LPCAT1 staining was increased in malignant as compared to benign esophageal tissue and was found in high intensity in 26.4% of 288 interpretable esophageal adenocarcinomas (EACs) and in 23.2% of 211 squamous cell carcinomas (ESCCs). Increased LPCAT1 staining was linked to undifferentiated tumor grading in both subtypes of EACs and ESCCs (*p* = 0.0273 and *p* = 0.0085).

**Conclusion:**

However, LPCAT1 was not associated with prognosis of EAC and ESCC patients (*p* = 0.6838 and *p* = 0.4695) and thus cannot be considered a prognostic biomarker in esophageal cancers.

## Introduction

Esophageal cancer is the eighth most common cancer in the world and one of the most malignant types of cancer (Song et al. 2014). The 5-year survival rate for all patients with esophageal cancer is 10%, which is related to the fact that there are no adequate mechanisms for early detection and treatment (Song et al. 2014). The discovery of new markers that could be used to predict tumor behavior or personalized therapy in individual patients is of great importance.

Certain processes in the cell, such as motility, growth, differentiation, and proliferation, which are ultimately also important for tumor cells, are influenced by changes in lipid metabolism (Santos und Schulze 2012). In addition, changes in superficial membrane potential and phospholipid composition are associated with malignancy (Dobrzyńska et al. 2005), and alterations of membrane lipid levels can also influence cell proliferation and viability (Preetha et al. 2005). Lysophosphatidylcholine acyltransferase 1 (LPCAT1) plays a key role in the synthesis of various individual phosphatidylcholine samples, which are important components of cell membranes and lipoproteins. (Kent 2005). LPCAT1 plays an important role in important physiological processes in the body such as the production of surfactants in the lungs (dipalmitoylphosphatidylcholine) (Bridges et al. 2010; Chen et al. 2006; Nakanishi et al. 2006), in the non-inflammatory platelet-activation factor remodeling pathway (Harayama et al. 2008) and in the retinal photoreceptor homeostasis (Chen et al. 2006).

Overexpression of LPCAT1 has been found in several malignancies, such as colon cancer. Dueke et al. (1996) showed that the amount of phospholipids in colon cancer tissue is greatly increased and increased synthesis of membrane phospholipids is required for rapid growth during tumor development (Dueke et al. 1996). In addition, analyzes from colon cancer cell lines have shown a significant growth advantage when overexpressing LPCAT1 (Mansilla et al. 2009). In esophageal cancer, one transcriptome analysis suggested an upregulation of LPCAT1 (Warnecke-Eberz et al. 2016). To further evaluate the potential of LPCAT1 as a prognostic biomarker in esophageal cancer, we analyzed a tissue microarray (TMA) containing more than 600 esophageal cancer specimens. In summary, our data identify an overexpression of LPCAT1 in a subset of esophageal cancers with undifferentiated tumor grading. However, since LPCAT1 expression was unrelated to prognosis of patients, LPCAT1 expression cannot be considered as a prognostic biomarker in esophageal cancers.

## Materials and methods

### Esophageal cancer TMA

A TMA was constructed from cancer tissues after radical esophagectomies from 359 esophageal adenocarcinoma and 254 esophageal squamous cell carcinoma patients treated at the Department of General, Visceral and Thoracic Surgery at the University Medical Center Hamburg-Eppendorf in a period from 1997 to 2018. The 8th edition of the TNM classification for esophageal cancer was used for staging. Follow-up data were available for 359 EAC and 254 ESCC patients with a median follow-up of 17.3 and 12.2 months (range 0–208 and 0–191 months). A 2-year follow-up was available for 39% of the patients and a 5-year follow-up for 26% of the patients. Our results show an overall 5-year survival of 35% for EACs and 15% for SCCs, which is in line with survival rates from the literature (Pennathur et al. 2013; Siegel et al. 2012). All esophagus specimens were analyzed according to a standard procedure, including complete embedding of the entire esophagus for histological analysis. The TMA manufacturing process was described earlier in detail (Mirlacher und Simon 2010). In short, one 0.6 mm core was taken from a representative tissue block from each patient. The tissues were distributed among 2 TMA blocks. For internal controls, each TMA block also contained various control tissues, including normal esophageal tissue. The study was approved by the Ethics commission Hamburg, and conducted in accordance with the Declaration of Helsinki. Usage of routinely archived formalin fixed leftover patient tissue samples for research purposes by the attending physician is approved by local laws and does not require written consent (HmbKHG, §12,1).

### Immunohistochemistry

Freshly cut TMA sections were immunostained in 1 day and in one experiment. Slides were deparaffinized and exposed to heat-induced antigen retrieval for 5 min in an autoclave at 121 °C in pH 7.8 Tris–EDTA-Citrate buffer. Primary antibody specific for LPCAT1 (rabbit, ProteinTech; at 1/1350 dilution) was applied according to the manufacturer´s directions. Bound antibody was then visualized using the EnVision Kit (Dako, Glostrup, Denmark). LPACT1 staining was homogenous in the analyzed tumor samples and staining intensity was thus semiquantitatively assessed in the following two categories: low and high immunostaining. Number of stained cells were counted in each tissue core (0.6 mm diameter), representing approximately one 40× high power field (HPF). Intensity was grouped in relation to the count distribution quantities according to the median into “low” (*n* < median) and “high” (*n* ≥ median). The method of classification was previously described (Hanke et al. 2015; Melling et al. 2019).

### Statistical analysis

Statistical calculations were performed with JPM 9 software (SAS Institute Inc., NC, USA). Contingency tables and the chi-squared test were performed to search for associations between molecular parameters and tumor phenotype. Survival curves were calculated according to Kaplan–Meier. The Log-Rank test was applied to detect significant survival differences between groups. Cox proportional hazards regression analysis was performed to test the statistical independence and significance between pathological, molecular, and clinical variables. Logistic regression was used to quantify the area under receiver-operator curve (ROC).

## Results

### Technical aspects

A total of 288 of 359 (80.2%) EACs and 211 of 254 (83.1%) ESCCs were interpretable for IHC analysis. Reasons for non-informative tissue cores included a lack of tissue or absence of unequivocal cancer cells on individual TMA spots.

### IHC of LPCAT1

LPCAT1 expression was generally cytoplasmic and occasionally showed a granular pattern. Intensity of LPCAT1 expression was increased in malignant as compared to benign esophageal tissue. Twenty benign tissues were analyzed. These specimens were retrieved from non-malignant esophagectomy specimens and served as control tissues for IHC staining, together with various other control tissues. None of the benign specimens showed high-intensity staining.

**Fig. 1:**
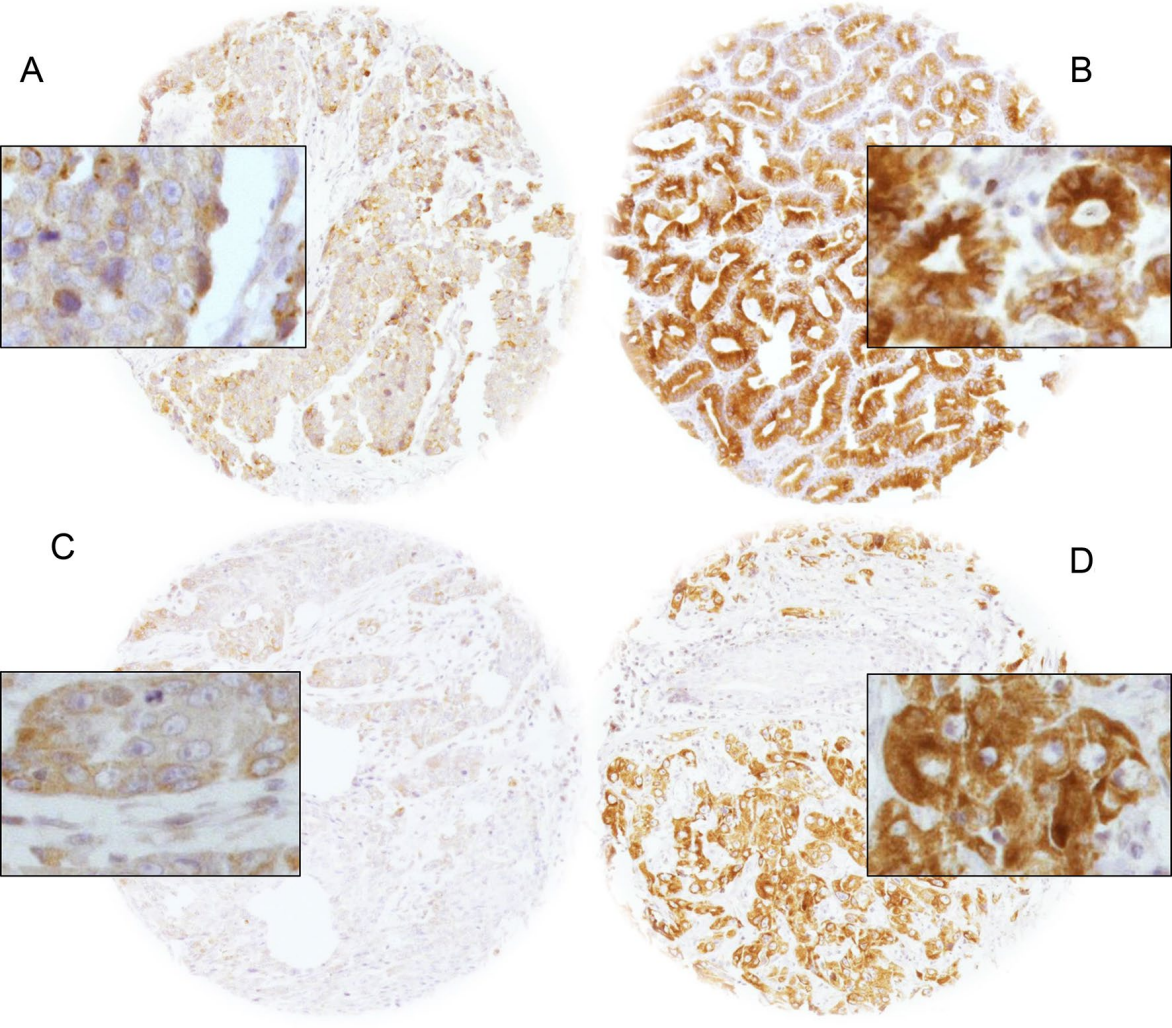
Immunohistochemical images of LPCAT1 staining. Images of low and high LPCAT1 expression in EACs (**A** and **B**) and ESCCs (**C** and **D**)

Representative images of LPCAT1 immunostaining are shown in Fig. 1.

### Association of LPCAT1 expression with tumor phenotype and prognosis of patients

**Fig. 2:**
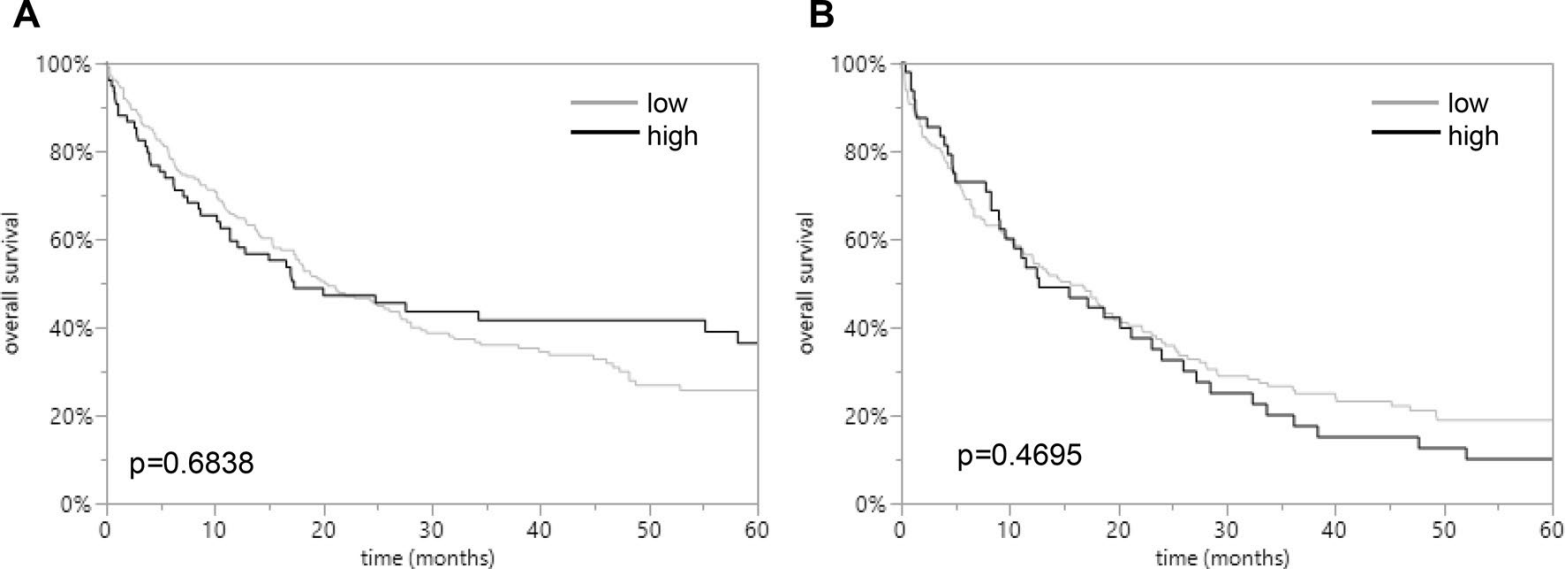
Prognostic impact of LPCAT1 expression in esophageal cancers. Relationship of LPCAT1 immunostaining intensity with overall survival in EACs (*p* = 0.6838; **A**) and ESCCs (*p* = 0.4695; **B**).

Increased LPCAT1 expression was linked to undifferentiated tumor grading in both subtypes of EACs and ESCCs (*p* = 0.0273 and *p* = 0.0085). The clinico-pathological parameters relative to LPCAT1 expression are shown in Tables 1 and 2. LPCAT1 staining was not associated with prognosis of EAC and ESCC patients (*p* = 0.6838 and *p* = 0.4695; Fig. 2).

**Table 1:** Clinico-pathological parameters relative to LPCAT1 IHC results in EACs

Parameter	Immunostaining			
	Evaluable (*n*)	Low (%)	High (%)	*p* value
Tumors	288	73.61	26.39	
Age group				0.598
< 65 years	98	75.51	24.49	
> 65 years	190	72.63	27.37	
Sex				0.3824
Male	244	74.59	25.41	
Female	44	68.18	31.82	
Tumor stage				0.0241
pT1	59	57.63	42.37	
pT2	34	76.47	23.53	
pT3	173	78.03	21.97	
pT4	20	80	20	
UICC stage				0.0043
I	58	55.17	44.83	
II	38	84.21	15.79	
III	164	76.83	23.17	
IV	26	80.77	19.23	
Grading				0.0273
G1	14	57.14	42.86	
G2	107	72.9	27.1	
G3	158	77.22	22.78	
G4	5	20	80	
R status				0.3235
R0	209	72.25	27.75	
R1	71	76.06	23.94	
R2	3	100	0	
pN category				0.1229
N0	88	27.14	41.33	
N1	50	19.05	13.33	
N2	64	24.29	17.33	
N3	83	29.52	28	
M status				0.3708
M0	262	72.9	27.1	
M1	26	80.77	19.23

**Table 2:** Clinico-pathological parameters relative to LPCAT1 IHC results in ESCCs

Parameter	Immunostaining			
	Evaluable (*n*)	Low (%)	High (%)	*p* value
Tumors	211	76.78	23.22	
Age group				0.5792
< 65 years	76	75	25	
> 65 years	134	78.36	21.64	
Sex				0.9408
Male	154	77.27	22.73	
Female	56	76.79	23.21	
Tumor stage				0.3838
pT1	36	86.11	13.89	
pT2	39	79.49	20.51	
pT3	123	73.98	26.02	
pT4	13	69.23	30.77	
UICC stage				0.6182
I	48	83.33	16.67	
II	54	75.93	24.07	
III	99	75.76	24.24	
IV	9	66.67	33.33	
Grading				0.0085
G1	3	100	0	
G2	131	83.21	16.79	
G3	76	65.79	34.21	
G4	0	0	0	
R status				0.1189
R0	154	79.87	20.13	
R1	47	65.96	34.04	
R2	8	87.5	12.5	
pN category				0.8271
N0	90	75.56	24.44	
N1	51	74.51	25.49	
N2	41	80.49	19.51	
N3	27	81.48	18.52	
M status				0.3416
M0	202	77.72	22.28	
M1	8	62.5	37.5

## Discussion

The results of our study do not support LPCAT1 immunostaining as a prognostic marker in esophageal cancer.

In the present immunohistochemical study, LPCAT1 immunostaining was increased in malignant as compared to benign esophageal tissue. High LPCAT1 immunostaining was detectable in 26.4% and 23.2% of the interpretable EACs and ESCCs. These results are generally in line with one earlier transcriptome analysis, which suggested an upregulation of LPCAT1 in both histological subtypes of esophageal cancers (Warnecke-Eberz et al. 2016).

The fact, that LPCAT1 expression was enhanced in malignant as compared to benign esophageal cancer may suggest a relevant role of LPCAT1 during esophageal tumorigenesis. This assumption matches several earlier studies implicating tumor relevant functional consequences of alterations in the lipid metabolism (Furuta et al. 2010; Santos und Schulze 2012; Menendez 2010; Menendez und Lupu 2007; Suburu und Chen 2012). Thus, activation of lipid biosynthesis and lipid remodeling has been suggested as a common feature of cancer cells and overexpression of several proteins involved in lipid metabolism, such as fatty acid-binding protein (Morgan et al. 2008), fatty acid synthase (Flavin et al. 2010a, b; SHAH et al. 2006), Caveolin-1 (Freeman et al. 2012) or fatty acid elongase 7 (Tamura et al. 2009) have been associated with tumorigenesis. Since LPCAT1 overexpression is also observed in aggressive forms of a broad variety of other cancer types (Uehara et al. 2016; Shida-Sakazume et al. 2015; Diefenbach et al. 2006) a general role of this protein during tumor progression has been suggested. Based on its molecular function as a key enzyme of lipid synthesis in the Land’s cycle, it is believed that LPCAT1 upregulation reflects the increased demand for lipid-depending cellular structures such as membranes and fatty acids in rapidly proliferating tumor cells (Currie et al. 2013). This is also supported by a study demonstrating that inhibition of enzymes of the Land’s cycle limits the growth of cancer cells and reduces tumorigenesis in various tumor cell models (Flavin et al. 2010a, b).

The results of our study identify LPCAT1 as a further protein of the lipid metabolism, which is involved in esophageal carcinogenesis.

It was another aim of this study to analyze the association of LPCAT1 expression with tumor phenotype and prognosis of patients. Increased LPCAT1 expression was significantly linked to undifferentiated tumor grading in both subtypes of EACs and ESCCs. These observations again, suggest a relevant role of LPCAT1 in esophageal tumorigenesis. However, although there was a trend of increased LPCAT1 immunostaining in unfavorable esophageal tumor phenotype, LPCAT1 expression was not prognostically relevant. Thus, our study does not support LPCAT1 expression as a potential relevant biomarker in esophageal cancers.

In summary, our data identify an overexpression of LPCAT1 in a subset of esophageal cancers with undifferentiated tumor grading. However, since LPCAT1 expression was unrelated to prognosis of patients, LPCAT1 cannot be considered as a prognostic biomarker in esophageal cancers.
